# Genetics of Ischaemic Stroke among Persons of Non-European Descent: A Meta-Analysis of Eight Genes Involving ∼ 32,500 Individuals

**DOI:** 10.1371/journal.pmed.0040131

**Published:** 2007-04-24

**Authors:** Roshan Ariyaratnam, Juan P Casas, John Whittaker, Liam Smeeth, Aroon D Hingorani, Pankaj Sharma

**Affiliations:** 1 Imperial College Cerebrovascular Research Unit (ICCRU), Department of Clinical Neuroscience, Hammersmith Hospitals and Imperial College, London; 2 Department of Epidemiology and Population Health, London School of Hygiene and Tropical Medicine; 3 Centre for Clinical Pharmacology, University College, London; Western General Hospital, United Kingdom

## Abstract

**Background:**

Ischaemic stroke in persons of European descent has a genetic basis, but whether the stroke-susceptibility alleles, the strength of any association, and the extent of their attributable risks are the same in persons of non-European descent remains unanswered. Whether ethnicity itself has a relevant or substantial contribution on those effect estimates is controversial. Comparative analyses between the ethnic groups may allow general conclusions to be drawn about polygenic disorders.

**Methods and Findings:**

We performed a literature-based systematic review of genetic association studies in stroke in persons of non-European descent. Odds ratios (ORs) and 95% confidence intervals (CIs) were determined for each gene–disease association using fixed and random effect models. We further performed a comparative genetic analysis across the different ethnic groups (including persons of European descent derived from our previous meta-analysis) to determine if genetic risks varied by ethnicity. Following a review of 500 manuscripts, eight candidate gene variants were analysed among 32,431 individuals (12,883 cases and 19,548 controls), comprising mainly Chinese, Japanese, and Korean individuals. Of the eight candidate genes studied, three were associated with ischaemic stroke: the *angiotensin I converting enzyme (ACE)* insertion/deletion (I/D) polymorphism with a mean OR of 1.90 (95% CI 1.23–2.93) in the Chinese and 1.74 (95% CI 0.88–3.42) in the Japanese; the summary OR for the C677T variant of *5,10-methylenetetrahydrofolate reductase (MTHFR)* was 1.18 (95% CI 0.90–1.56) in Chinese and 1.34 (95% CI 0.87–2.06) in Koreans; and the pooled OR for the *apolipoprotein E (APOE)* gene was 2.18 (95% CI 1.52–3.13) in Chinese and 1.51 (95% CI 0.93–2.45) in Japanese. Comparing the commonly investigated stroke genes among the Asian groups against studies in persons of European descent, we found an absence of any substantial qualitative or quantitative interaction for ORs by ethnicity. However, the number of individuals recruited per study in the studies of persons of non-European descent was significantly smaller compared to studies of persons of European descent, despite a similar number of studies conducted per gene.

**Conclusions:**

These data suggest that genetic associations studied to date for ischaemic stroke among persons of non-European descent are similar to those for persons of European descent. Claims of differences in genetic effects among different ethnic populations for complex disorders such as stroke may be overstated. However, due to the limited number of gene variants evaluated, the relatively smaller number of individuals included in the meta-analyses of persons of non-European descent in stroke, and the possibility of publication bias, the existence of allele variants with differential effects by ethnicity cannot be excluded.

## Abstract 2

This meta-analysis found that genetic associations so far studied for ischemic stroke among non-Europeans are similar to those found for persons of European descent.

## Editors' Summary

**Background.:**

A stroke occurs when the blood supply to part of the brain is interrupted, either because a blood vessel supplying the brain becomes blocked or because one ruptures. Strokes are a substantial cause of death and disability worldwide, with most of the burden affecting people living in developed countries. Most strokes fall into a category termed ischemic stroke. This type is caused by blockages in the blood vessels supplying the brain, which can happen when there is a buildup of fatty deposits or clots within the blood vessels. Many of the risk factors for this particular type of stroke are affected by an individual's behavior, including for example smoking, high blood pressure, diabetes, inactivity, and so on. In addition, variations in an individual's genetic makeup might affect his or her chance of having a stroke. Previous research studies have shown that variants in many different genes are likely to be involved in determining the overall risk of having a stroke, each variant contributing in a small way to the risk.

**Why Was This Study Done?:**

The group performing this study had previously carried out a systematic review of existing research, looking specifically at the genetics of ischemic stroke among people of European origin (often called “Caucasians”). However, it was not obvious whether the genetic risk factors for stroke they found would be the same for people from a different ethnic background. Therefore the research group wanted to find out what the genetic risk factors were for stroke among people of non-European origin and to compare these findings with those of their previous systematic review. This research might help to find out whether the genetic risk factors for stroke were different in people from different parts of the world.

**What Did the Researchers Do and Find?:**

As a starting point, these researchers wanted to find all the different studies that had already been carried out examining the effect of genetic risk factors on stroke among people of non-European origin. To do this, searches were carried out of electronic databases using a particular set of terms. All resulting studies that involved genetic research in people of non-European origin and in which strokes were confirmed by brain scanning were then evaluated in more detail. The findings of different studies were combined if at least three studies were available for the same genetic variant. Eventually 60 studies were found that looked at the association between eight specific gene variants and stroke. The only data that could be included in a combined analysis came from Chinese, Japanese, and Korean populations. Three of the eight gene variants were associated with an increased risk of stroke. Those three gene variants were *ACE* I/D (a variant in the gene coding for angiotensin 1-converting enzyme, which is involved in controlling blood pressure); a variant in *MTHFR* (which codes for the enzyme methylenetetrahydrofolate reductase, and which converts certain amino acids within cells); and a variant in the gene *APOE,* which codes for a protein that plays a role in breaking down fats. The researchers then compared their findings from this study with the findings of a previous systematic review they had carried out among people of European origin. Overall, each gene studied seemed to have a similar effect in the different populations, with the exception of *APOE,* which seemed to be associated with stroke in the Asian studies but not in the studies from people of non-European origin. The researchers also found that generally the Asian studies suggested a slightly greater effect of each gene variant than the studies in people of non-European origin did.

**What Do These Findings Mean?:**

These findings suggest that, with the possible exception of *APOE,* similar gene variants play a role in determining stroke risk in people of European origin and Asian populations. Although generally the studies examined here suggested a slightly greater effect of these gene variants in Asian populations, this is not necessarily a real finding. This greater effect may just be due to small-study bias. Small-study bias describes the observation that small research studies are more likely to produce a false positive result than are large research studies. Therefore, future studies that examine the genetic basis of stroke should recruit much larger numbers of participants from populations made up of people of non-European origin than has previously been the case.

**Additional Information.:**

Please access these Web sites via the online version of this summary at http://dx.doi.org/10.1371/journal.pmed.0040131.

- Health Encyclopedia entry on stroke from NHS Direct (UK National Health Service patient information)
- Stroke Information page from the National Institute of Neurological Disorders and Stroke (provided by the US National Institutes of Health)
- The Stroke Association, a UK charity funding this study
- Information from the World Health Organization on the distribution and burden of stroke worldwide
- The WHO has a world atlas of heart disease and stroke

## Introduction

The incidence and standardised mortality ratios for stroke are known to vary between different ethnic groups [1], which might reflect genetic or nongenetic differences. Sporadic ischaemic stroke in persons of European descent has a multifactorial aetiology [2–6], with evidence for a genetic basis [7–9].

However, whether stroke susceptibility alleles, their genetic effect sizes, or attributable risks are qualitatively (similarity of gene variants) or quantitatively (size of effects of such variants) homogenous across groups of differing ancestry [10] remains controversial [11].

We performed a systematic review of genetic association studies of sporadic ischaemic stroke in persons of non-European descent and compared the findings to a prior overview we conducted among persons of European descent [10], which allowed us to address both qualitatively and quantitatively the effect of ethnicity on ischaemic stroke, often regarded as a paradigm of the common multifactorial, polygenic disorders. This is, to our knowledge, the largest genetic meta-analysis conducted in persons of non-European descent for any disease.

## Methods

### Database Search

Electronic databases (Medline, http://www.ncbi.nlm.nih.gov/entrez/query.fcgi?DB=pubmed; EMBASE, http://www.embase.com; Google Scholar, http://scholar.google.com; and Yahoo, http://www.yahoo.com) were searched up to and including January 2005 for all genetic-association studies evaluating any candidate gene in stroke in persons of non-European descent. The medical subject headings, terms, and text words used for the search were: cerebrovascular disease, stroke, brain infarction, and cerebrovascular disorder, in combination with polymorphism, genotype, gene, allele, or mutation. The search results were limited to humans and all languages were included. The references of all the computer-identified publications were searched for any additional studies, and the related articles option (a feature available on Medline) was used to search for any further possible related articles. Another search to identify all previous genetic meta-analyses in stroke was also performed.

Studies were selected if they were conducted in persons of non-European descent, had neuroimaging (magnetic resonance imaging or computer tomography) confirmation of an ischaemic stroke diagnosis, and were analysed as a dichotomous trait. Studies were excluded if: (i) patients were under 18 years of age; (ii) they evaluated quantitative or intermediate phenotypes exclusively; or (iii) genotype frequency was not reported, in which case authors were contacted for this information where possible. For duplicate publications the smaller dataset was discarded.

### Statistical Analyses

Data were analysed using software for preparing and maintaining Cochrane reviews (Review Manager version 4.2.8, Cochrane Collaboration, http://www.cc-ims.net/RevMan) and Comprehensive Meta Analysis version 2.2.023 (Biostat, http://www.biostat.org).

For each single nucleotide polymorphism where data were available from at least three studies, a meta-analysis was carried out as described previously [10]. The genetic models evaluated are given in Table 1 and were based mostly on those used in the primary studies. For each variant a pooled odds ratio (OR) was calculated using random effects models, along with 95% confidence intervals (CI), to measure the strength of the association. For comparability with our previous report in persons of European descent, we used the same genetic model of inheritance for each gene variant when necessary [10]. Heterogeneity was assessed by the DerSimonian and Laird *Q* test [12], and *I* ^2^ was used as a measure to describe the percentage of variability in point estimates that was due to heterogeneity rather than sampling error [13]. For assessment of small-study bias, we used the funnel plot and the Egger regression asymmetry test [14].

**Table 1:**
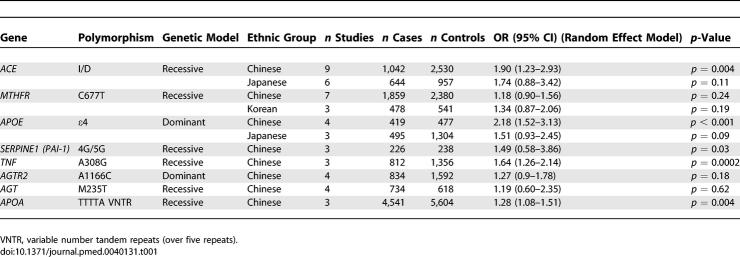
Summary of Candidate Genes in Ischemic Stroke Evaluated in Persons of Non-European Descent

The effects of ethnicity in gene–stroke associations were evaluated using stratified subgroups according to ethnicity. The *I* ^2^ test was used to evaluate the presence of heterogeneity of genetic size between groups of differing ancestry. For the group of persons of European descent the OR for each gene variant analysed was obtained from our previous work [10]. Differences between populations of non-European descent and persons of European descent in the allele frequencies from control samples across the different candidate genes were evaluated by means of a Chi^2^ analysis.

While numerous tools exist to help reviewers assess the quality of randomised trials, assessment of study quality in the synthesis of observational and genetic research is more controversial. Most experts working in the field generally advise avoiding the use of quantitative scoring of study quality in meta-analyses, as such an approach has not been validated and can itself introduce bias. One of the reasons, highly relevant in genetic studies, is the often inadequate reporting that constrains the elaboration of such scores to judge the study quality. In addition, there is little or no empirical evidence to guide reviewers on which aspects of study design have an influence on the results obtained. Study size is, however, a widely used proxy for study quality. We therefore undertook a sensitivity analysis, in which concordance of the overall result for each meta-analyses was conducted with the results after excluding the largest study. The key issue in the assessment of stroke as an outcome is classification as ischaemic or haemorrhagic. To minimize outcome misclassification, we therefore restricted our analyses to those studies in which neuroimaging was used to classify the stroke as ischaemic.

## Results

### Candidate Genes in Persons of Non-European Descent

A total of 500 manuscripts were identified in our initial search, of which 60 met the inclusion criteria including studies among Chinese, Japanese, Koreans, African Americans, and South Asians. Of these, only studies among the former three Asian groups had sufficient data to allow inclusion into a meta-analysis. We analysed eight candidate genes. Table 1 shows the candidate genes studied by ethnic group.

The Chinese population was by far the most extensively studied across the various candidate genes (Table 2). The second most-studied ethnic group was the Japanese. Overall, the two most extensively studied candidate genes in persons of non-European descent were *angiotensin I converting enzyme (ACE)* and *5,10-methylenetetrahydrofolate reductase (MTHFR),* followed by *apolipoprotein E (APOE)* and *SERPINE1 (plasminogen activator inhibitor-1 [PAI-1]*) (Table 2).

**Table 2:**
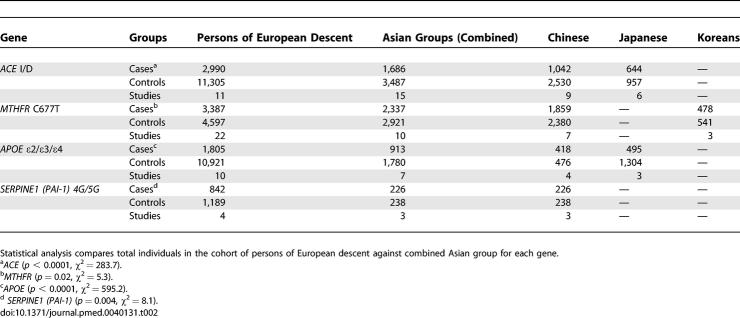
Comparative Total Number of Cases, Controls, and Studies by Ethnicity

**Figure 1:**
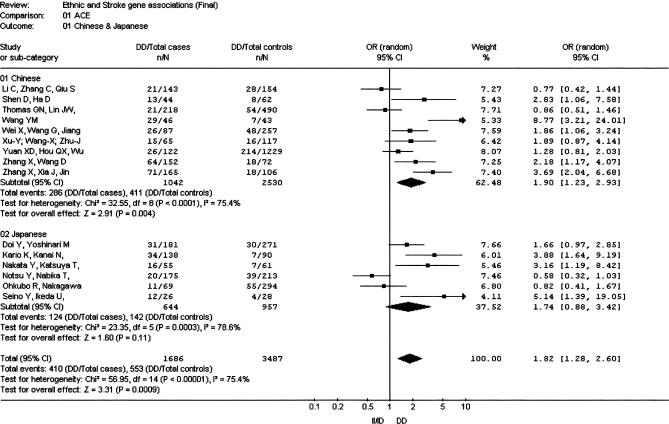
Meta-Analysis of Studies of the *ACE* I/D Polymorphism and Risk of Stroke in Chinese and Japanese

The insertion/deletion polymorphism of *ACE* (*ACE* I/D) was the most investigated polymorphism in all three ethnic groups (a total of 3,572 Chinese individuals, 1,601 Japanese individuals, and 2,750 Korean individuals). The overall OR for the nine studies in the Chinese population was 1.90 (95% CI 1.23–2.93) (Figure 1) [15–23]. There was evidence for heterogeneity (*I* ^2^ = 75.4; *p*_HET_ < 0.0001) among studies within this ethnic grouping. For the six Japanese studies [24–29] the OR was 1.74 (95% CI 0.88–3.42) (Figure 1). Heterogeneity of the OR was also observed within this grouping (*I* ^2^ = 78.6; *p*_HET_ = 0.0003). We identified four Korean studies [30–33], but they were not included in the meta-analysis because of suspicion of duplicate publication. The overall OR in the Asian group combined (Chinese and Japanese) was 1.82 (95% CI 1.28–2.60). Significant interstudy heterogeneity was observed (*I* ^2^ = 75.4; *p*_HET_ < 0.0001). The funnel plot was asymmetric, and the Egger test was significant (*p* = 0.027).

**Figure 2:**
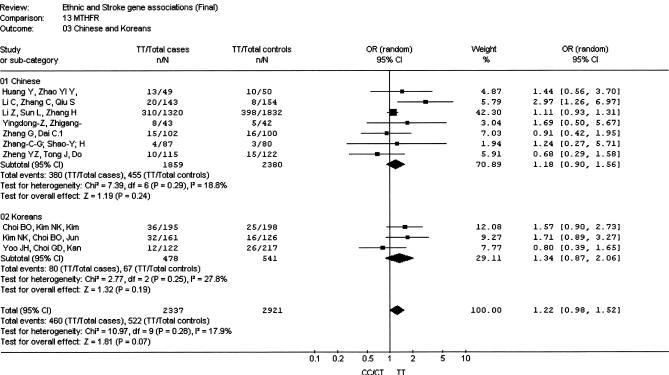
Meta-Analysis of Studies of the *MTHFR* C677T Variant and Stroke Risk in Chinese and Koreans

A total of seven studies (1,859 cases and 2,380 controls) among Chinese populations evaluating the C677T variant in the *MTHFR* gene were identified [22,34–39]. A summary OR of 1.18 (95% CI 0.90–1.56) was observed for individuals homozygous for the T allele compared with C-allele carriers (i.e., CT + CC) (Figure 2). No significant interstudy heterogeneity was observed (*I* ^2^ = 18.8; *p*_HET_ = 0.29), and although the results were dominated by the relatively large study of Li et al. [35], excluding this study did not substantially alter the results. We identified three Korean studies [40–42] with a total of 478 cases and 541 controls providing an OR of 1.34 (95% CI 0.87–2.06) with no significant interstudy heterogeneity (*I* ^2^ = 27.8; *p*_HET_ = 0.25). A pooled analysis of Chinese and Koreans samples provided an overall OR of 1.22 (95% CI 0.98–1.52), and no evidence of heterogeneity was observed (*I* ^2^ = 17.9; *p*_HET_ = 0.28). No evidence of asymmetry was observed in the funnel plot, and the Egger test was not significant (*p* = 0.42).

**Figure 3:**
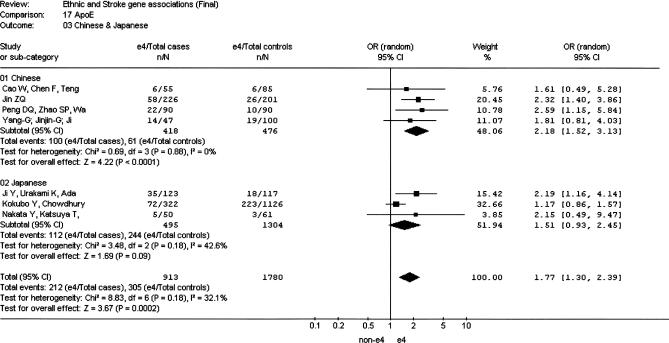
Meta-Analysis of Studies of the *APOE* ɛ2/ɛ3/ɛ4 Polymorphism and Risk of Stroke in Chinese and Japanese

A total of seven studies in Asians (four in Chinese [418 cases and 476 controls] [43–46] and three in Japanese [495 cases and 1,304 controls] [47–49]) evaluating the *APOE* ɛ4 polymorphism against the pooled ɛ2/ɛ3 were identified. The summary OR of the Chinese studies was 2.18 (95% CI 1.52–3.13) (Figure 3). No heterogeneity was observed (*I* ^2^ = 0; *p*_HET_ = 0.88). The pooled OR of the three Japanese studies [47–49] was 1.51 (95% CI 0.93–2.45). The overall OR in the seven Asian studies was 1.77 (95% CI 1.30–2.39), and although there was no formal statistical evidence of heterogeneity overall (*I* ^2^ = 32.1; *p*_HET_ = 0.18) and between groups (Figure 3), studies conducted in the Chinese population tended to obtain a higher OR than that observed in the Japanese population. Despite the small number of studies, evidence of asymmetry in the funnel plot was observed, and the Egger test suggested the presence of small-study bias (*p* = 0.08).

Although only three studies [50–52] in Chinese populations evaluated the pentanucleotide TTTTA repeat polymorphism of the *apoliproprotein A (APOA)* gene on ischaemic stroke with a pooled OR of 1.28 (95% CI 1.08–1.51; *I* ^2^ = 0%; *p*_HET_ = 0.39), the total number of cases and controls (4,541 and 5,604) was substantially higher than for all other gene variants. It was mainly due to the large case-control study by Sun et al. [52] in which a significant increase of risk of stroke was reported.

A number of other gene variants were studied in a smaller number of individuals (Table 1). Polymorphisms in the genes *SERPINE1 (PAI-1)* [53–55], *tumour necrosis factor-alpha (TNF)* [56–58], *angiotensin II type 1 receptor (AT1R)* [17,59–61], and *angiotensinogen (AGT)* [18,49,61,62] were all studied only in Chinese populations. Nominally significant ORs were observed for *TNF* and *SERPINE1 (PAI-1),* with the latter result consistent with previous results in persons of European descent 10. However, the number of studies for each genetic polymorphism was considerable smaller than that for other gene variants (Table 1). In addition, we could not discard the existence of some overlap among the studies that evaluated the *TNF* gene [56,57].

### Genetic Effect Sizes in Individuals of Differing Ancestry

For each of the major four major genes studied *(ACE, MTHFR, APOE,* and *SERPINE1 [PAI-1]),* the total number of cases studied were significantly greater in the samples of persons of European descent compared with the combined ethnic group (Table 2). In addition, the mean number of individuals per polymorphism studied was greater in the persons of European descent compared to the combined group of persons of non-European descent (Table 3). The only exception to this finding was for the *MTHFR* gene, for which the mean was boosted by one large study [35].

**Table 3:**
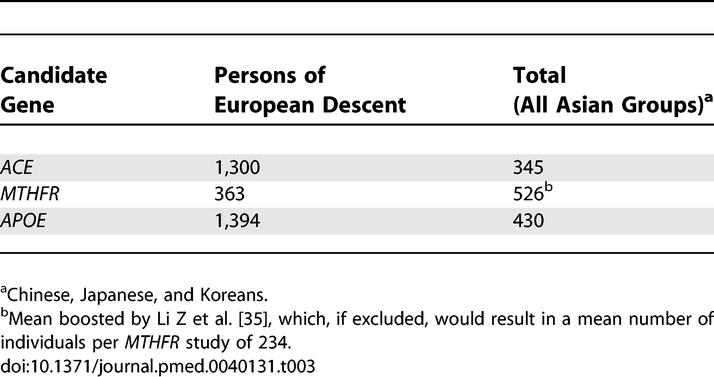
Mean Number of Individuals Studied by Gene according to the Ethnic Background

**Figure 4:**
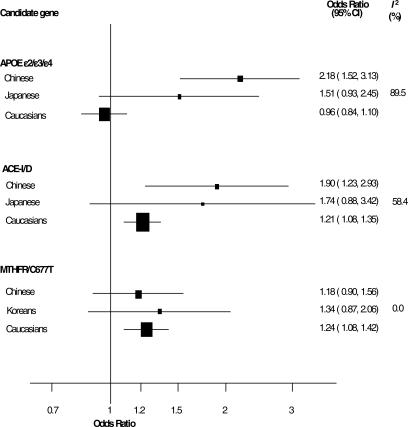
Comparison of ORs for Stroke for the Polymorphisms in *MTHFR, ACE,* and *APOE* Genes Respectively, among Individuals of Differing Ancestry *I^2^*, measure of heterogeneity

Of the genes studied, four *(ACE, MTHFR, APOE,* and *SERPINE1 [PAI-1])* had also been extensively studied in persons of European descent, which enabled us to compare their effects against our previous meta-analysis in persons of European descent (Table 4) [10]. With the exception of *ACE* I/D, there were no significant differences of the allele frequencies between persons of European descent and Asians. A quantitative analysis of OR of these genes shows that the effect sizes are broadly similar across the different ethnic groups apart from *APOE,* which has a null effect in persons of European descent but a significant association with stroke in the Chinese and Japanese groups (Figure 4).

**Table 4:**
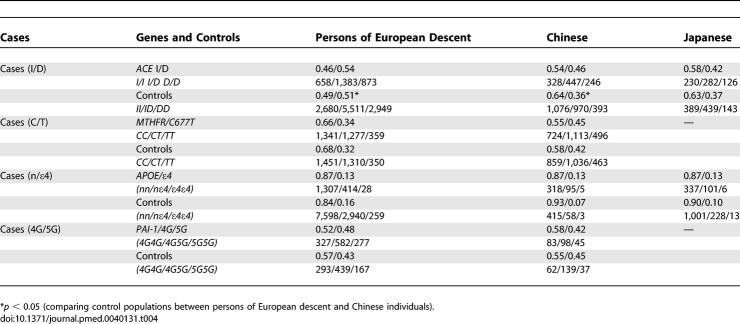
Allele and Genotype Frequencies by Ethnicity

## Discussion

Despite numerous attempts to address the question of genetic liability in sporadic ischaemic stroke in persons of non-European descent using association studies, the number of susceptibility genes and their relative risk remain unclear, mainly hampered by the lack of power of any individual study. The current meta-analysis suggests the existence of gene variants, similar to those evaluated in persons of European descent, associated with ischaemic stroke in persons of non-European descent, mostly represented by studies from populations of Asian background with scarce data from other ethnic groups.

Although some candidate gene variants showed a statistically significant increase in risk of stroke, the robustness of these results remains open to discussion. The observed genetic effect on stroke in the persons of non-European descent, especially in Chinese samples, tended to be higher than the effect observed in persons of European descent, as well as with that observed in other Asian groups such as the Japanese population. These findings, together with the usually small sample size of studies conducted in the persons of non-European descent, suggest publication bias as one possible explanation for the observed larger genetic effects in Chinese samples. In contrast to *APOE* and *ACE* genes, the results from the *MTHFR* C677T gene variant and stroke risk show an absence of heterogeneity among the different groups of persons of non-European descent studied, as well as between these groups and the genetic effect obtained in samples of persons of European descent [10]. The robustness of this association reported in the meta-analysis has received further support by a large study [63] in the Japanese population, published after our cutoff point for data collection, in which the *MTHFR* C677T gene variant was associated with a similar increase in risk of stroke to the one we report here.

A descriptive analysis of the most-studied gene variant indicated that with the exception of the *MTHFR* C677T gene variant, summary ORs in persons of non-European descent tend to be higher than those reported in persons of European descent, the effect mainly accounted for by the results in the Chinese population (Figure 4). While the results in samples of persons of European descent indicated a null effect of the *APOE* ɛ4 allele on stroke, the pooled OR from studies of Chinese people was substantially higher, and their CIs did not overlap (Figure 4). A similar although more attenuated difference between persons of European descent and Japanese was observed. Therefore, it is not surprising that when comparing the pooled results separately for the three groups, substantial heterogeneity was observed (*I* ^2^ = 89.5%). Interestingly, the genetic effect observed in the Chinese population was even higher than that observed in other Asian ethnic groups, such as the Japanese population, although some overlap of their CIs remains (Figure 4). A similar scenario, although less pronounced, was observed for the *ACE* I/D gene variant, in which studies conducted in the Chinese population showed a higher estimate of the effect than that observed in persons of European descent, which accounted for most of the heterogeneity observed among these ethnic groups (*I* ^2^ = 58.4%). In contrast, for the *MTHR* C677T variant, the effect estimate within the groups of non-European descent (Chinese and Koreans), as well as when compared with that in persons of European descent, was homogenous (*I* ^2^ = 0%; Figure 4).

Although the existence of genuine genetic heterogeneity in the Chinese population to explain their larger genetic effects is possible, the probable causal reasons to explain this finding may not be always directed toward the same direction (higher risk) for the different candidate genes. Equally likely is that, since association studies conducted in persons of non-European descent are smaller compared to persons of European descent, ethnicity may act as a proxy for the presence of small-study bias, whether it represents publication bias, poorer study quality, or greater random error. Study quality itself is difficult to measure, but heterogeneity forms one aspect of this measurement. Whether the positive gene–disease ORs observed here will reduce toward a null effect in very large studies conducted in different ethnic groups remains to be seen. A plausible scenario is that the genetic effect is indeed genuine but lower than it currently appears to be, such as the case of the *MTHFR* C677T, in which the summary OR in persons of non-European descent coincides with the result of the largest genetic association study.

The larger genetic effect observed in these candidate gene–stroke meta-analyses for Chinese studies, despite the smaller sample sizes, is in agreement with a recent systematic review of 13 gene–disease associations from a wide variety of complex disorders, in which gene effects in samples from Chinese people tended to be larger than those observed in non-Chinese Asian people, and more accentuated with studies conducted in persons of European descent. An interesting difference with that analysis is the case of the *MTHFR* C677T gene–coronary heart disease association in which the authors, in contrast to our findings, also observed a larger genetic effect in Chinese samples [64].

We are aware of one previous study that has attempted to address the question of ethnic differences in genetics of a wide variety of complex traits [11]. These investigators examined the genetic effects for 43 validated gene–disease associations across nearly 700 populations of various descents. Large variations in genetic effect sizes by ethnic group arose in only 14% of cases, suggesting the genetic markers for gene–disease associations may vary across populations, but the biological effects, at least for common diseases, is similar across the traditional ethnic boundaries [11]. Apart from the unusually large effect in Chinese samples, our results do provide some support to the notion of common, underlying biological causes across different ethnic groups for common disorders such as ischaemic stroke. As genotype frequencies do seem to differ to some extent by ethnic group (e.g., *ACE* I/D in persons of European descent versus Chinese individuals), the impact of similar genetic effects may be different in other ethnic populations.

Despite our attempt to evaluate the gene effects in all ethnic groups, we were limited by the fact that the vast majority of data in persons of non-European descent came from Chinese, Japanese, and Korean populations. The nature and extent of the genetic contribution to stroke and other multifactorial disorders in groups of differing ancestry should be considered an important endeavour for risk evaluation and primary prevention measure across all populations. First, evaluation of genetic effects across multiple ethnic backgrounds with substantial difference in cultural behaviour may prove to be the best scenario for the study of gene–environment interactions. Second, the presence of concordance of gene effects on disease risk, as well as on its effect on the direct intermediate phenotype, are strong tools to reliably identify putative functional variants. Third, since the uses of a genetic approach by using gene variants as tools of common intermediate phenotype is expected to provide great advantage in aetiological research, adequately powered genetic studies may help to unravel aetiological factors for the increasing public health disorders in persons of non-European descent such as stroke, which is rapidly becoming a major cause of mortality in Asia with over 1.6 million deaths in China alone in 2002 [65]. Certainly, such an enterprise would require extensive international collaboration among investigators in the field, since gene effects on disease are usually small. The emergence of recently established networks such as HuGENet (Human Genome Epidemiology, http://www.hugenet.org.uk) will prove to play an essential role for an adequate study of gene effects in persons of non-European descent [66].

As with all meta-analyses based on aggregate data, our results are dependent on published data. Although, as described above, efforts were conducted to limit small-study bias, (the most likely cause of which is publication bias), significant results of the test for small-study bias indicate that this bias has to be carefully considered..In addition, even ethnicity can be a difficult trait to define and sometimes is inconsistently reported in the literature. That the included studies have tended to come from their native countries, should minimize such misclassification. In addition, self-reported ethnic grouping, which is likely to take place in native countries, is known to be very reliable for categorization purposes [67]. Finally, for some genes our results are based on small numbers of publications, which limits the power of our tests for publication bias and heterogeneity.

We conclude that genetic associations so far studied for ischaemic stroke among persons of non-European descent are similar to those found for persons of European descent. However, all the published data on persons of non-European descent are largely derived from Chinese, Japanese, and Korean populations. To date, the evidence does not support ethnicity playing a major part in the genetic aetiology of common ischaemic stroke. However, people from a more diverse range of ethnic groups need to be recruited into genetic studies in much greater numbers than is currently the case.

## Abbreviations

CI: confidence interval
OR: odds ratio
